# A Silent Saddle Embolism: Syncope Without Dyspnea or Chest Pain

**DOI:** 10.7759/cureus.94747

**Published:** 2025-10-16

**Authors:** Sheel Vaniawala, Racquille Reid, Nikhil Sikha, Anita Sikha

**Affiliations:** 1 Internal Medicine, University of Miami Hospital and Clinics/Holy Cross Hospital, Fort Lauderdale, USA; 2 Internal Medicine, Trinity Health Oakland Hospital, Pontiac, USA; 3 Graduate Medical Education, University of Miami Miller School of Medicine, Fort Lauderdale, USA

**Keywords:** mechanical thrombectomy (mt), myocardial injury, pulmonary embolism (pe), right ventricular dysfunction, saddle embolus, syncope, troponin elevation

## Abstract

Pulmonary embolism (PE) is a potentially fatal diagnosis that may present without classic symptoms. We describe a rare presentation of a submassive saddle PE manifesting solely as syncope, without dyspnea or chest pain. The patient was found to have significant right ventricular dysfunction and myocardial injury with elevated troponin and was ultimately diagnosed with a large saddle PE and successfully treated with catheter-directed thrombectomy and anticoagulation. This case underscores the importance of echocardiographic RV assessment and early imaging in syncope workup to avoid delayed or missed PE diagnoses.

## Introduction

Pulmonary embolism (PE) is the third most common cause of cardiovascular mortality after myocardial infarction and stroke, with an annual incidence of approximately 60-70 per 100,000 individuals worldwide [1]. Despite advances in diagnosis and treatment, PE remains underdiagnosed due to its highly variable presentation, ranging from asymptomatic cases to sudden death [2]. Syncope is reported in 10-17% of patients with acute PE and is associated with larger clot burden, right ventricular (RV) dysfunction, and worse short-term prognosis [3]. While chest pain and dyspnea are the most recognized presenting features, isolated syncope without accompanying respiratory symptoms is relatively uncommon and may delay diagnosis [4].

The pathophysiology of syncope in PE is multifactorial, involving acute RV failure, decreased cardiac output, arrhythmogenesis, and transient cerebral hypoperfusion [5]. Diagnosis is challenging in these patients since the absence of typical features may divert attention toward more common causes of syncope, such as arrhythmias, vasovagal episodes, or structural cardiac disease. In addition, troponin elevation in this setting may lead clinicians to suspect acute coronary syndrome rather than PE [6].

Management of submassive PE, defined by RV dysfunction and biomarker elevation in the absence of hemodynamic collapse, remains controversial, with options including anticoagulation, systemic thrombolysis, catheter-directed thrombolysis, and mechanical thrombectomy [7]. Mechanical thrombectomy has emerged as a valuable alternative in patients with high clot burden and intermediate-high-risk features, especially when systemic thrombolysis carries increased bleeding risk [8].

We present a case of a middle-aged male with no prior medical history who developed syncope as the sole presenting symptom of a submassive saddle PE, ultimately managed with mechanical thrombectomy. This case highlights the diagnostic challenges of atypical presentations and underscores the importance of considering PE in patients with unexplained syncope and myocardial injury markers.

## Case presentation

A 68-year-old male with no known past medical history presented to the emergency department after a witnessed syncopal episode. The event occurred at home, where the patient suddenly lost consciousness with only preceding lightheadedness. He denied preceding chest pain, dyspnea, palpitations, or other prodromal symptoms. He recovered spontaneously within one minute and was brought in by family.

On arrival, he was alert and oriented. Initial vital signs revealed a blood pressure of 118/76 mmHg, a heart rate of 96 beats per minute, a respiratory rate of 18 breaths per minute, an oxygen saturation of 94% on room air, and a temperature of 36.8°C. Physical examination showed no jugular venous distension, clear lungs to auscultation, and regular heart sounds without murmurs. No focal neurological deficits were appreciated.

**Figure 1:**
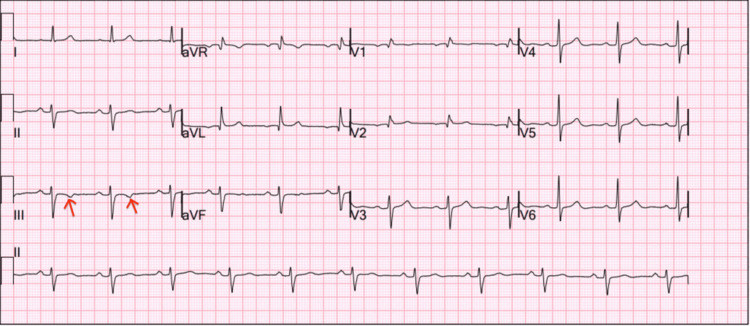
Twelve-lead ECG obtained on admission demonstrating sinus rhythm with isolated T-wave inversion in lead III (arrows) This nonspecific repolarization abnormality may be seen in acute PE and correlates with right heart strain. PE, pulmonary embolism

ECG demonstrated normal sinus rhythm with nonspecific T-wave inversions in the anterior leads (Figure 1). Laboratory studies were notable for elevated cardiac biomarkers. Complete laboratory results are summarized in Table 1.

**Table 1:** Admission laboratory values All values are presented with reference ranges. Notable abnormalities included elevated troponin-I and B-type natriuretic peptide, indicating myocardial strain, as well as mild anemia.

Test	Result	Reference range
Hemoglobin	12.1 g/dL	13.5-17.5 g/dL
White blood cell count	9.8 × 10⁹/L	4.0-11.0 × 10⁹/L
Platelets	245 × 10⁹/L	150-400 × 10⁹/L
Sodium	138 mmol/L	135-145 mmol/L
Potassium	3.9 mmol/L	3.5-5.1 mmol/L
Creatinine	1.2 mg/dL	0.6-1.3 mg/dL
Blood urea nitrogen	21 mg/dL	7-20 mg/dL
Troponin-I (high sensitivity)	0.34 ng/mL	<0.04 ng/mL
B-type natriuretic peptide	432 pg/mL	<100 pg/mL

**Figure 2:**
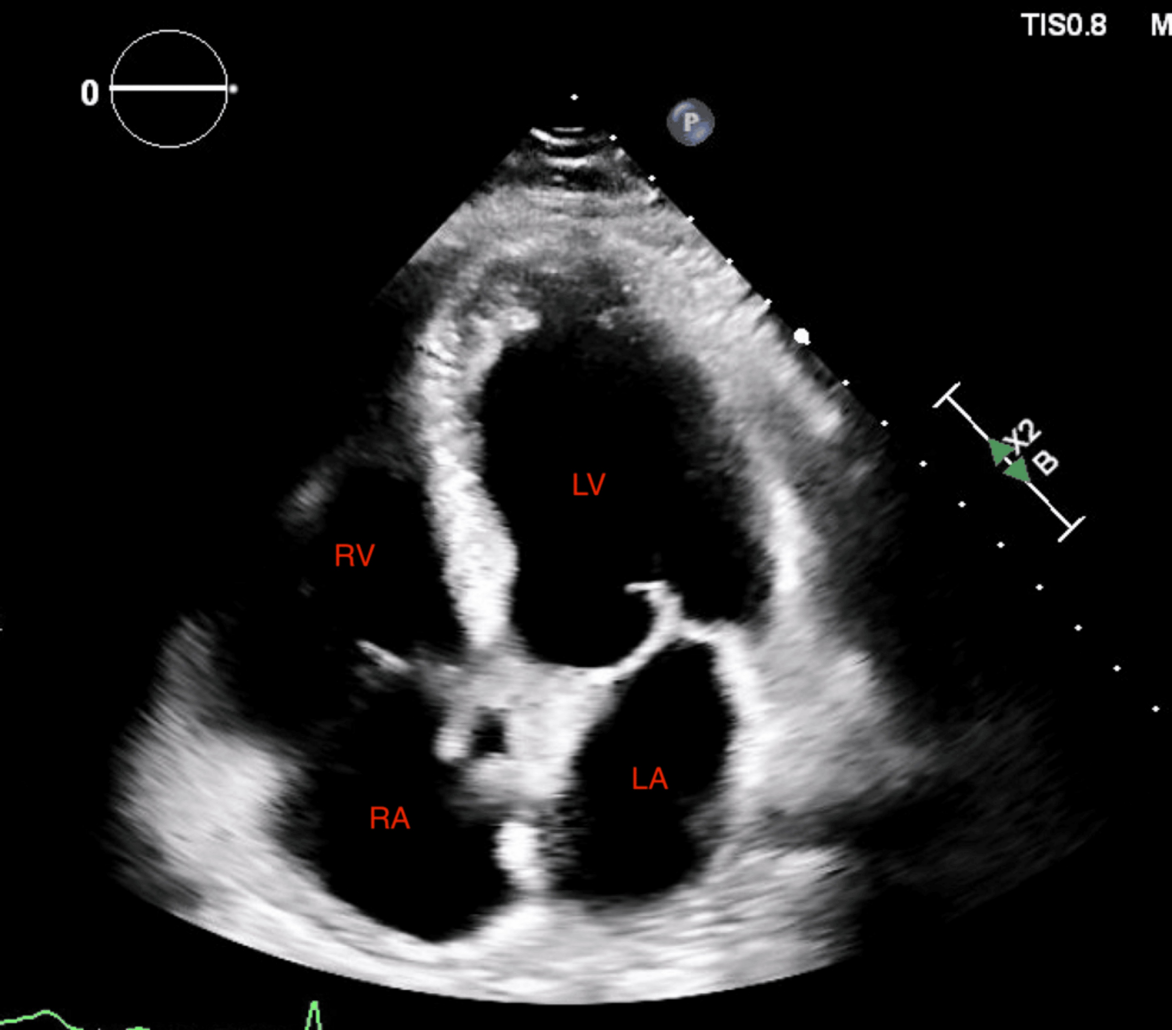
Transthoracic echocardiogram (apical four-chamber view) showing a severely enlarged RV (outlined) with reduced systolic function compared to the LV RV dilation is consistent with RV pressure overload in the setting of PE. LA, left atrium; LV, left ventricle; PE, pulmonary embolism; RA, right atrium; RV, right ventricle

**Figure 3:**
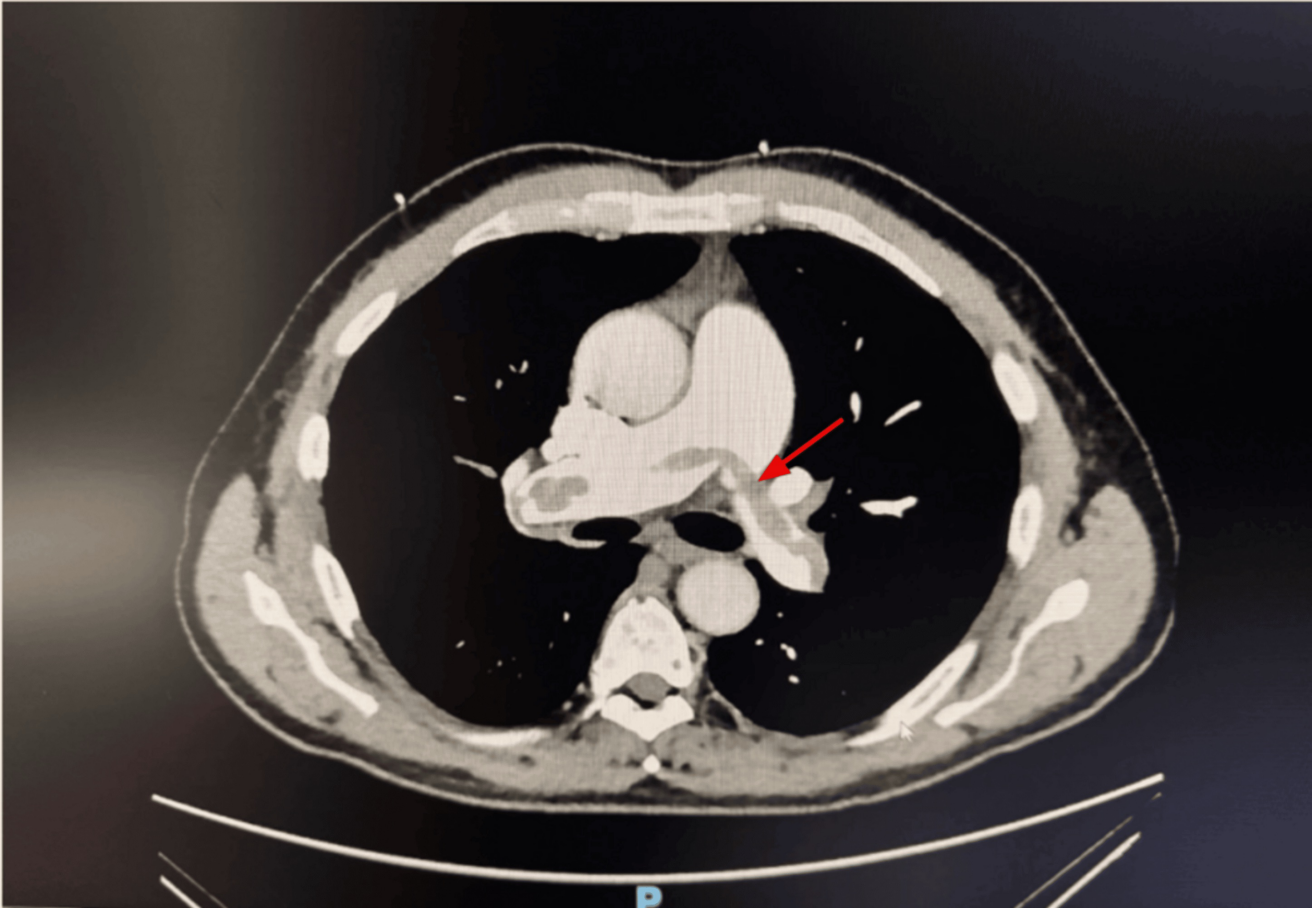
CTA of the chest with contrast demonstrating a large saddle PE (arrow) extending into the right and left main pulmonary arteries CTA, CT angiography; PE, pulmonary embolism

**Figure 4:**
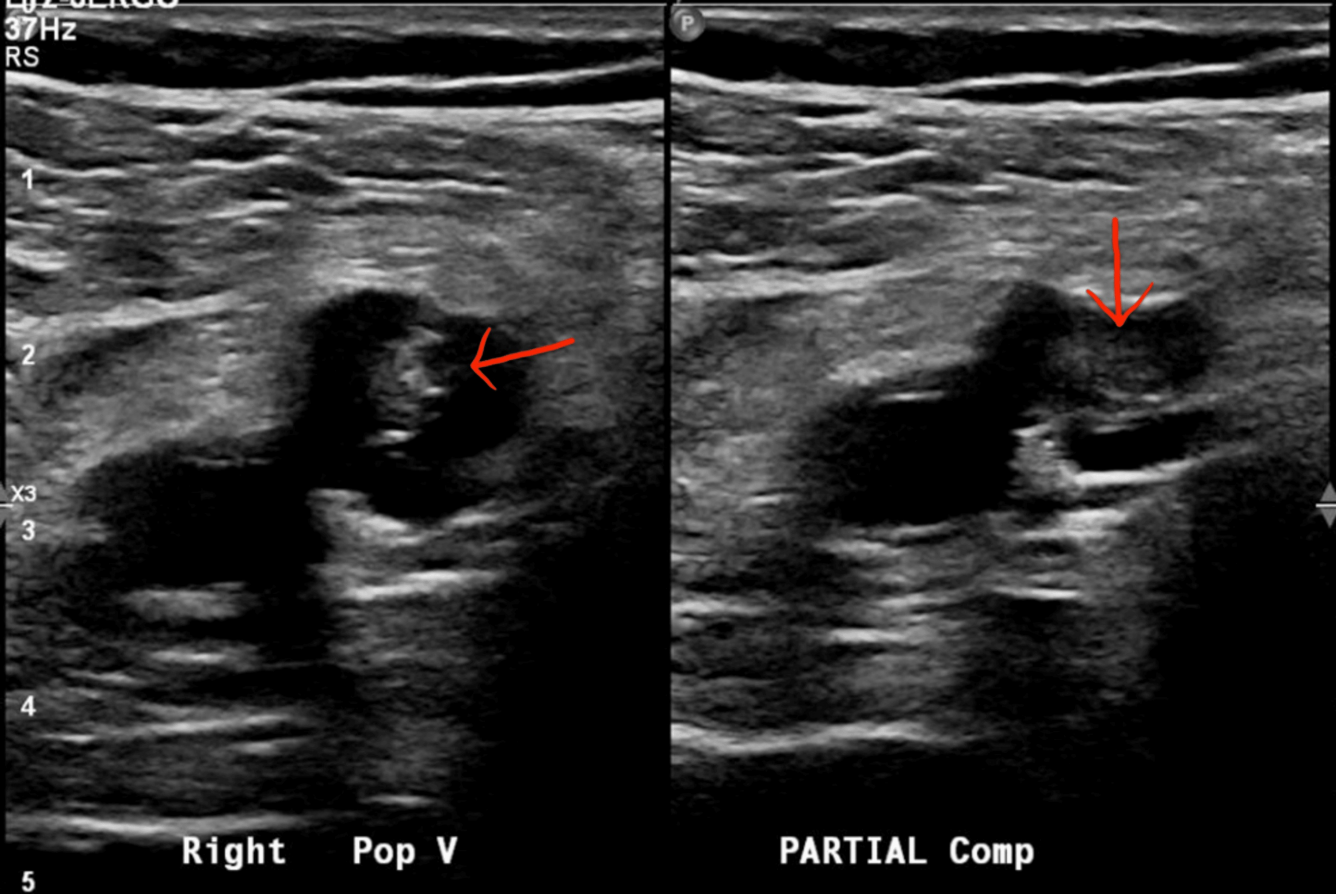
Duplex Doppler ultrasound of the right lower extremity demonstrating echogenic thrombus within the right popliteal vein (arrow) The absence of compressibility and visualization of intraluminal material confirm the presence of acute DVT, serving as a potential source for PE. DVT, deep venous thrombosis; PE, pulmonary embolism

Given the unexplained syncope with elevated troponin and BNP, a transthoracic echocardiogram was obtained, which revealed an ejection fraction of 55-60% with severe RV dilation, an RV/LV ratio >1, and moderate pulmonary hypertension (Figure 2). CT pulmonary angiography subsequently demonstrated a large saddle PE with extension into bilateral main pulmonary arteries (Figure 3). Lower-extremity Doppler ultrasound revealed deep venous thrombosis in bilateral popliteal veins (Figure 4).

The patient was started on intravenous unfractionated heparin. After multidisciplinary discussion, given the intermediate-high-risk features (RV dysfunction and biomarker elevation) and high clot burden, the patient underwent catheter-directed mechanical thrombectomy. The procedure achieved a significant reduction in clot burden with improved pulmonary artery flow. Post-procedure, the patient’s hemodynamics remained stable, and his oxygenation improved. He was transitioned to apixaban for long-term anticoagulation and discharged home on day five with outpatient cardiology and hematology follow-up.

## Discussion

PE remains a significant cause of morbidity and mortality, with presentations ranging from asymptomatic to life-threatening hemodynamic compromise. Our case highlights a submassive PE with RV dysfunction successfully managed with thrombectomy. Submassive PE, defined by evidence of RV strain in the absence of systemic hypotension, poses a diagnostic and therapeutic challenge, as prompt recognition and intervention are crucial to prevent progression to hemodynamic instability or death [1].

Diagnostic challenges

Early diagnosis of submassive PE can be difficult because patients may present with nonspecific symptoms such as dyspnea, chest pain, or syncope, as observed in our patient. Imaging modalities such as CT pulmonary angiography remain the gold standard, while echocardiography can detect RV dysfunction and elevated pulmonary pressures, aiding risk stratification [4]. Biomarkers, including troponin and BNP, can also serve as adjunctive tools to identify myocardial strain, although their interpretation requires careful clinical correlation [6]. In our patient, the combination of elevated troponin and echocardiographic RV dysfunction reinforced the need for urgent intervention despite hemodynamic stability.

Treatment challenges and current practices

Management of submassive PE is nuanced. While anticoagulation is standard, patients with evidence of RV dysfunction and biomarker elevation may benefit from advanced interventions, including systemic thrombolysis, catheter-directed thrombolysis, or mechanical thrombectomy [7]. Thrombolysis, although effective, carries a significant bleeding risk, especially in older patients or those with comorbidities. Catheter-based or surgical thrombectomy offers the advantage of rapid clot removal with potentially lower hemorrhagic complications. In our case, thrombectomy was chosen due to the patient’s intermediate-high risk profile and contraindications to systemic thrombolysis, aligning with recent expert recommendations [8].

Expert perspectives and recommendations

Experts emphasize individualized risk assessment for submassive PE. According to the European Society of Cardiology guidelines, patients with RV dysfunction and elevated biomarkers should be monitored closely in a critical care setting, with escalation to reperfusion therapy considered if clinical deterioration occurs [1]. Multidisciplinary PE response teams have been shown to improve outcomes by providing rapid expert assessment and facilitating timely intervention [9]. Our management strategy reflects these principles, demonstrating the importance of early recognition, risk stratification, and timely referral for thrombectomy in selected patients.

Comparisons with literature

Similar cases in the literature underscore the efficacy of mechanical thrombectomy in submassive PE. Studies report significant hemodynamic improvement, reduced RV strain, and favorable short-term outcomes, particularly in patients at high risk for bleeding complications from systemic thrombolysis [8,10]. Our patient’s rapid symptomatic improvement and normalization of RV function are consistent with these observations, supporting the growing role of catheter-based interventions in appropriately selected cases.

Limitations and future directions

While mechanical thrombectomy shows promise, randomized controlled trials directly comparing it with systemic thrombolysis are limited [11]. Long-term outcomes, optimal patient selection criteria, and cost-effectiveness remain areas for further research. Clinicians must also weigh the benefits of early intervention against procedural risks and the availability of institutional expertise.

## Conclusions

This case highlights the diagnostic and therapeutic challenges in submassive PE with RV dysfunction. Early recognition, risk stratification, and timely intervention are critical to improving patient outcomes. Mechanical thrombectomy can be a safe and effective option in selected patients, particularly when systemic thrombolysis is contraindicated or high-risk. Multidisciplinary collaboration and adherence to guideline-directed care ensure optimal management, while further studies are needed to refine patient selection and assess long-term outcomes of catheter-based interventions.
